# Rhombohedral Li_1+x_Y_x_Zr_2-x_(PO_4_)_3_ Solid Electrolyte Prepared by Hot-Pressing for All-Solid-State Li-Metal Batteries

**DOI:** 10.3390/ma13071719

**Published:** 2020-04-06

**Authors:** Qinghui Li, Chang Xu, Bing Huang, Xin Yin

**Affiliations:** 1 College of Electrical and Information Engineering, Hunan University, Changsha 410082, China; B12090021@hnu.edu.cn; 2 State Key Laboratory of New Ceramics and Fine Processing, School of Materials Science and Engineering, Tsinghua University, Beijing 100084, China; MSExuchang@outlook.com; 3 School of Materials Science and Engineering, Tianjin University, Tianjin 300072, China

**Keywords:** NASICON, hot pressing, interfacial resistance, Li-ion conductivity, all-solid-state batteries

## Abstract

NASICON-type solid electrolytes with excellent stability in moisture are promising in all-solid-state batteries and redox flow batteries. However, NASIOCN LiZr_2_(PO_4_)_3_ (LZP), which is more stable with lithium metal than the commercial Li_1.3_Al_0.3_Ti_1.7_(PO_4_)_3_, exhibits a low Li-ion conductivity of 10^−6^ S cm^−1^ because the fast conducting rhombohedral phase only exists above 50 °C. In this paper, the high-ionic conductive rhombohedral phase is stabilized by Y^3+^ doping at room temperature, and the hot-pressing technique is employed to further improve the density of the pellet. The dense Li_1.1_Y_0.1_Zr_1.9_(PO_4_)_3_ pellet prepared by hot-pressing shows a high Li-ion conductivity of 9 × 10^−5^ S cm^−1^, which is two orders of magnitude higher than that of LiZr_2_(PO_4_)_3_. The in-situ formed Li_3_P layer on the surface of Li_1.1_Y_0.1_Zr_1.9_(PO_4_)_3_ after contact with the lithium metal increases the wettability of the pellet by the metallic lithium anode. Moreover, the Li_1.1_Y_0.1_Zr_1.9_(PO_4_)_3_ pellet shows a relatively small interfacial resistance in symmetric Li/Li and all-solid-state Li-metal cells, providing these cells a small overpotential and a long cycling life.

## 1. Introduction

Rechargeable Li-metal batteries with a high-voltage cathode and Li-metal as an anode have much higher energy density than conventional rechargeable Li-ion batteries with a graphite anode. However, the Li-metal batteries have serious safety issues, because of the fast lithium dendrite formation and growth during charge; moreover, the limited chemical/electrochemical stability of the liquid organic electrolyte when the cell is operated at high temperatures and high voltages can result in the failure or even the explosion of the batteries [1,2,3,4]. Compared with liquid organic electrolytes, solid electrolytes have high mechanical strength and good thermal stability, and some solid electrolytes have similar Li-ion conductivities to the liquid electrolytes [5,6,7]. Moreover, the good wetting ability of the solid electrolyte by the metallic lithium anode can reduce or even remove the lithium dendrite formation in all-solid-state Li-metal batteries [8,9,10]. Therefore, developing all-solid-state Li-metal batteries by replacing the liquid electrolyte with a high Li-ion conductive solid-state electrolyte is one of the most effective strategies to improve the safety and energy density of the batteries.

Research on solid electrolytes is focusing on polymer electrolytes, ceramic electrolytes, and polymer/composite electrolytes. Ceramic electrolytes usually have higher Li-ion conductivities than polymer and polymer/composite electrolytes because of the slow chain motion of the polymer at room temperature [11,12,13]. The ceramic electrolytes also have much better mechanical strength than the polymer electrolyte. Two kinds of ceramic electrolytes, including oxide and sulfide ceramics, are promising candidates in all-solid-state Li-metal batteries. Compared with the sulfide ceramics, the oxide ceramics show a larger electrochemical window, a much better stability in air, and a much lower cost. Oxide garnet-type (e.g., Li_7_La_3_Zr_2_O_12_) [14,15,16,17,18,19], NASICON-type (e.g., Li_1+x_MxTi_2-x_(PO_4_)_3_, M = Al, Ge) [20,21,22,23,24,25,26], perovskite-type (e.g., Li_3x_La(_2/3)−x_□_(1/3)−2x_TiO_3_) [27,28,29,30,31], and antiperovskite-type electrolytes (e.g., Li_2_OHX, X = Cl, Br) [32,33,34,35,36,37,38] have been reported to have high Li-ion conductivities at room temperature because of the suitable Li-ion transport channel inside the framework. Garnet and antiperovskite electrolytes are reported to be unstable in moist air, and the reaction between them and moisture destroys the structure, reduces the Li-ion conductivity of the solid electrolyte, and significantly increases Li-ion resistance across the interface [39,40,41]. Perovskite electrolytes and commercial NASICON electrolytes contain Ti^4+^ ions which are unstable at low voltages less than 2 V, although both of them are reported to be stable in water [42]. Replacing the Ti^4+^ by other stable metal ions may improve the stability of the perovskite and NASICON electrolytes with lithium metal. However, NASICON-type LiZr_2_(PO_4_)_3_ presents a complex polymorphic behavior upon synthesis [43,44]. Four different crystalline forms (α’ and α, β’ and β phase), depending on different annealing procedures, have been reported [45,46]. The rhombohedral α phase with a space group R-3c prepared at high temperature transfers to the triclinic α’ phase at temperatures < 60 °C. The orthorhombic β phase prepared at low temperature transfers to the monoclinic β’ phase with a space group of P2_1_/n at 300 °C. Only the rhombohedral α phase of LiZr_2_(PO4)_3_ is reported to have a high ionic conductivity above 10^−5^ S cm^−1^ at 50 °C. However, the rhombohedral phase changes to the triclinic phase at room temperature, which has a much lower Li-ion conductivity of 10^−7^ S cm^−1^.

In this work, we investigated the structural, chemical, and electrochemical stabilities of Y^3+^-doped LiZr_2_(PO_4_)_3_ by changing the Y^3+^ concentration; the structural stability of the sample was also characterized with XRD from −30 to 150 °C. We also tried different sintering technologies to study the influence of density on the electrochemical performance of the pellet. Different cells, including the symmetric Li/Li cell and Li/LiFePO4 cell, were prepared to study the performance of the NASICON electrolyte in a battery and also to study ionic transport at the electrolyte/electrode interface. Y^3+^ doping in LiZr_2_(PO_4_)_3_ was effective in improving the structural stability at a wide temperature range. A high Li-ion conductivity of 9 × 10^−5^ S cm^−1^ at room temperature was obtained for the sample prepared by hot pressing, which is much higher than the conductivity of the pellet prepared by spark plasma sintering [47]. The XPS depth profiles revealed that a passivation layer with a thickness of less than 100 nm formed on the surface of the pellet, which improved the cycling of the symmetric cell.

## 2. Experimental

For preparing Li_1+x_Y_x_Zr_2−x_(PO_4_)_3_ (0 ≤ x ≤ 0.2), stoichiometric LiNO_3_ (20% excess, Sinopharm, 4N, Beijing, China), Y(NO_3_)_3_⋅6H_2_O (Sinopharm, 4N, Beijing, China), Zr(NO_3_)_4_⋅5H_2_O (Sinopharm, AR, Beijing, China), (NH_4_)_2_HPO_4_ (Sinopharm, 4N, Beijing, China) and citric acid (Aladdin, AR, Shanghai China) raw materials were dissolved in 100 mL deionized water and thoroughly mixed with a magnetic stirrer at 80 °C to obtain a homogeneous solution. A precursor was obtained after evaporating the solution at 70 °C. The obtained mixture was heated at 900 °C for 6 h to remove the absorbed water and decompose the phosphates and nitrates in the precursor. The precursor was then ball-milled in a planetary mill (Fritsch, Pulverisette 4, Pittsboro, United States) with anhydrous isopropanol at 300 rpm min^−1^ for 8 h, and the powders were dried and subsequently pressed into a pellet with a diameter of 10 mm. Li_1+x_Y_x_Zr_2−x_(PO_4_)_3_ pellets were fired at 1150 °C for 12 h [48,49,50]. A Pt crucible was used to prepare the samples in a box furnace. Highly dense pellets were achieved by hot-pressing (ZT-40-20YB, Shanghai Chen Hua Electric Furnace Co. Ltd., Shanghai, China) at 1100–1250 °C for 3 h with an applied pressure of 50 Mpa. The finally obtained pellets were cut into pieces with a thickness of 500 μm for further electrochemical testing. The density of the pellet was obtained by dividing the mass by the volume of the pellet. Powder X-ray diffraction (XRD) patterns for the as-prepared samples were characterized with a Bruker-AXS D8-A25 (Billerica, United States) Advance diffractometer using Cu_Kα_ radiation. The XRD pattern was collected from 10 to 60 degrees, with a step size of 0.02 degree; the lattice parameters of the obtained samples were obtained with JADE software. The phase compositions of the NASICON pellets with different Y^3+^ concentrations were obtained by refining the XRD data of the samples with Fullprof software (Version: Sep-2019); the lattice parameters, the shape parameters of the peaks, and the full width at half maximum (FWHM) parameters were refined. Scanning electron microscopy (SEM) and Energy-dispersive X-Ray spectroscopy (EDS) images were examined by a field-emission scanning electron microscopy (SEM, FEI Quanta 650, Hillsboro, United States). The impedance spectra of Li_1+x_Y_x_Zr_2−x_(PO_4_)_3_ (0.1 ≤ x ≤ 0.2) were collected with a precision impedance analyzer (Agilent 4294A, Santa Clara, United States) in the temperature range of 298–423 K and fitted by the equivalent circuit (R_g_CPE_g_)(R_gb_CPE_gb_)CPE. The applied frequency was 110 MHz–40 Hz with an AC amplitude of 10 mV. X-ray photoelectron spectrometer (XPS) spectra (ESCALab250Xi, ThermoFisher Scientific, Waltham, United States) of Li, O, Zr, P elements were characterized to monitor the interfacial reaction of Li/NASICON using a VG ESCALAB MKII spectrometer with an Al Ka monochromatic X-ray source. An Ar ion gun was used to etch at a sputtering rate of 25 nm min^−1^.

LY_0.1_ZP pellets prepared by hot pressing were used for all the battery testing. For the symmetric cell testing, two lithium foils (0.5 cm^2^) were put on both sides of the pellet, and the cell was cycled at a current density of 0.05 mA cm^−2^ with a duration time of 1 h at 60 °C. The symmetric Li/LY_0.1_ZP/Li cell after cycling for 80 h was disassembled in the glovebox, and the small pieces from the broken LY_0.1_ZP pellet were transferred to the SEM equipment for SEM and EDS mapping measurements. The lithium foil had a thickness of 100 μm and a surface area of 0.5 cm^2^. One layer of Ni foam was put on top of the lithium foil to maintain good contact between lithium foil and the pellet. For the all-solid-state battery, the LiFePO_4_ cathode was prepared by mixing LiFePO_4_, carbon, PEO, and LiTFSI salt in acetonitrile with a weight ratio of 70:10:13:7. The cathode was dried in the oven at 80 °C for 24 h to remove the liquid acetonitrile. The slurry was coated on the aluminum foil by a doctor blade, and the loading of the active material was 3 mg cm^−2^. The Li/LiFePO_4_ cell was cycled between 3.8 and 2.8 V at 60 °C. In all-solid-state Li/LiFePO_4_ cells, the lithium and Li_1+x_Y_x_Zr_2−x_(PO_4_)_3_ pellets were separated by a solid polymer to reduce the interfacial resistance. The polymer was prepared by mixing PEO (M*_w_*: 600,000) and Lithium bis(trifluoromethanesulfonyl)imide (LiTFSI) with an EO/Li^+^ ratio of 10 in acetonitrile. The solution was stirred overnight and cast on a polytetrafluoroethylene dish. The polymer electrolyte was dried at 60 °C for 48 h. The impedances of the symmetric cell and the all-solid-state cell were measured in an Autolab working station (Herisau, Switzerland) with frequencies from 1 MHz to 1Hz. The cycling of the battery testing was conducted in land battery test systems (CT2001A, Wuhan, China).

## 3. Results and Discussion

**Figure 1:**
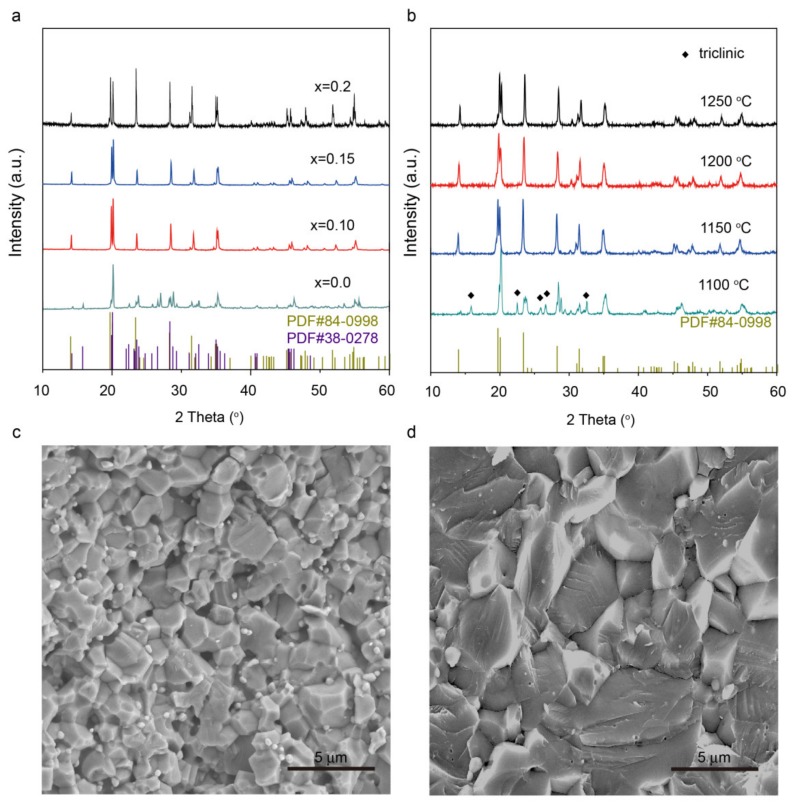
(**a**) XRD patterns of Li_1+x_Y_x_Zr_2−x(_PO_4_)_3_ (0 ≤ x ≤ 0.2) electrolyte fired at 1150 °C for 12 h. (**b**) XRD patterns of LY_0.1_ZP treated by hot-pressing (HP) at 1100–1250 °C. The cross-section SEM images of LY_0.1_ZP treated by (**c**) regular sintering and (**d**) hot-pressing. PDF #84-0998 and #38-0278 in (**a**) correspond to the rhombohedral and triclinic structure of LiZr_2_(PO_4_)_3_, respectively.

The XRD patterns of as-synthesized powders in a regular box furnace were compared in Figure 1a; dopant-free LiZr_2_(PO_4_)_3_ exhibited a monoclinic phase at room temperature, which is consistent with a previous report [46]. The pellet with a rhombohedral structure was obtained with Y^3+^ doping; all the diffraction peaks for Li_1+x_Y_x_Zr_2−x_(PO_4_)_3_ (x = 0.1 and 0.15) samples can be indexed to R-3c group. Some impurities existed in the sample with x = 0.2. The lattice parameters of the sample increased with increasing Y^3+^ concentration because of the larger ionic size of Y^3+^ relative to Zr^4+^, as shown in Figure S1. The XRD results of Li_1.1_Y_0.1_Zr_1.9_(PO_4_)_3_ prepared by hot-pressing at 1100–1250 °C for 3 h are shown in Figure 1b. Hot-pressing treatment did not change the rhombohedral phase when the pellets were fired at temperatures above 1150 °C. The small shoulder at 20 degrees in the sample with x = 0.15 and 0.2 indicates there may be minimal triclinic LiZr_2_(PO_4_)_3_ in the pellet. The sample with x = 0.1 could be fitted well with the rhombohedral phase, and the sample with x = 0.15 and 0.2 could be refined well with the mixed rhombohedral and the triclinic phases (Figure S2). The contents of the triclinic phases in the samples with x = 0.15 and 0.2 were 3.5% and 29.2%, respectively. Increasing Y^3+^ ions also increased the triclinic phase, which reduces the ionic conductivity of the NASICON electrolyte. The SEM image in Figure 1c shows that Li_1+x_Y_x_Zr_2−x_(PO_4_)_3_ with x = 0.1 (denoted as LiY_0.1_ZP) was composed of particles with an average grain diameter of 2 µm. The relative density of LY_0.1_ZP pellets prepared by regular sintering was 76% and is further improved to 94% by hot-pressing (HP) (Figure S3). The pellet prepared by hot pressing showed some transgranular fractures, indicating the grain–boundary of the pellet has a higher mechanical strength. No closed pores and fewer grain boundaries can be observed in LY_0.1_ZP sintered by HP at 1200 °C for 3 h (Figure 1d and Figure S4). The reduced porosity and grain boundaries could improve Li-ion transfer at the grain boundaries.

**Figure 2:**
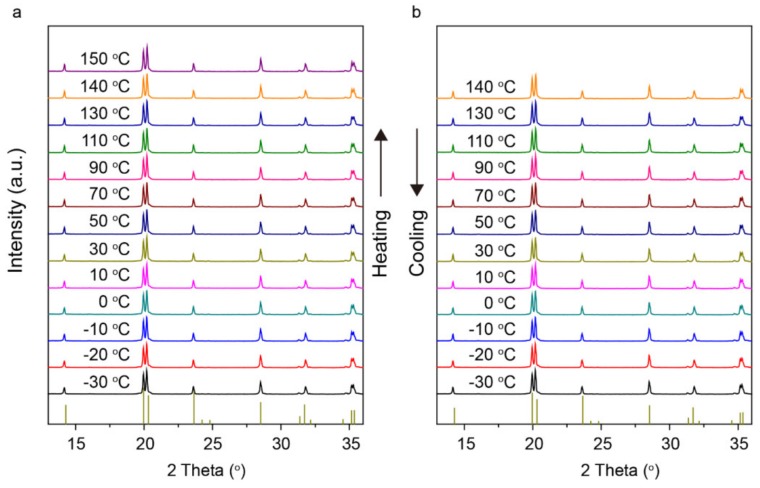
XRD patterns for the as-prepared LY0.1ZP electrolyte from −30 to 150 °C: (**a**) during the heating and (**b**) the cooling process.

To better understand the phase stabilization by Y^3+^ doping, phase composition vs. temperature was collected by in-situ powder X-ray diffraction (XRD). It can be seen from the XRD patterns in Figure 2a,b that characteristic peaks of the monoclinic phase in NASICON did not appear during heating from −30 to 150 °C and during the cooling process. It is evidenced that a high level of consistency with the characteristic rhombohedral *α* phase was obtained in a wide temperature range. There was no clear lattice parameter change during the heating and cooling process. The unit cell parameters of the as-prepared sample before heating (V = 1480.00 Å^3^, a = 8.8169 Å, c = 21.9834 Å) and the heated samples (e.g. 150 °C, V = 1480.08 Å^3^, a = 8.8111 Å, c = 22.01374 Å) are almost identical. The lattice stabilization with only minor thermal expansion, for LY_0.1_ZP, may benefit from a stronger bond strength of Y^3+^-O^2−^. It is reported that Y^3+^ doping can reduce the M1 site interstitial space and increase the volume of M2’ [43]; in our case the lattice parameter *a* = 8.8111 Å for LY_0.1_ZP shrank a little compared to LiZr_2_(PO_4_)_3_ at 150 °C (8.8549 Å); this local lattice distortion gives rise to the enhancement of ionic conductivity. According to the literature, the α→α’ transition of LiZr_2_(PO_4_)_3_ is generally at 50 °C [44]. In our case, the pure rhombohedral α phase of LY_0.1_ZP retained the structure at room temperature without the first-order transition. There is no phase deterioration, and the first-order transition from a rhombohedral α phase to a monoclinic phase occurs even at −30 °C, showing an effective structural stabilization by Y^3+^ doping.

**Figure 3:**
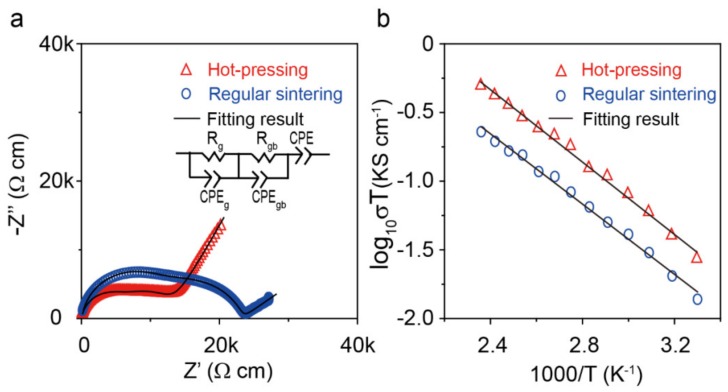
(**a**) Li-ion conductivities of LY_0.1_ZP at room temperature. The applied frequency was 110 MHz–40 Hz. and (**b**) Arrhenius plots of LY_0.1_ZP prepared by regular sintering and hot-pressing; the Arrhenius plots are based on the total impedances of the pellets.

The impedance spectra of the Li_1+x_Y_x_Zr_2−x_(PO_4_)_3_ (0.1 ≤ x ≤ 0.2) pellets prepared by regular sintering and hot pressing are shown in Figure 3 and Figure S5. The curves show two semicircles at the high and low frequency, which correspond to Li-ion transfer in the bulk and grain boundary of the pellet. The bulk and grain-boundary resistance could be obtained by fitting the experimental data with a conventional equivalent circuit consisting of (R_g_CPE_g_)(R_gb_CPE_gb_)CPE. For the samples prepared by regular sintering, the LY_0.1_ZP pellet showed the highest bulk and total Li-ion conductivity of 4.5 × 10^−5^ S cm^−1^, respectively; the samples with x = 0.15 and 0.2 have a lower ionic conductivity than the sample with x = 0.1, which may be caused by the impurities in the samples with increasing Y^3+^ concentration. The total Li-ion conductivity of the pellet prepared by hot-pressing was improved to 9 × 10^−5^ S cm^−1^ at room temperature. Hot-pressing significantly reduced the grain boundary resistance of the LY_0.1_ZP pellet, and the total ionic conductivity of the pellet prepared by hot-pressing is two orders of magnitude higher than that of LiZr_2_(PO_4_)_3_ without doping. Enhancement in Li-ion conductivity can be explained by (1) the increasing concentration of lithium ions and (2) the shrinkage of the M1 cavity and the expansion of the M2 space with Y^3+^ doping, which provide a shorter Li1- Li1’ hopping distance and lower bond strength for Li2 along the Li1-Li2 pathway [43]. Li-ion conductivities as a function of temperature in the range of 30–150 °C were collected, and a linear Arrhenius behavior was observed (Figure 3b); the rhombohedral LY_0.1_ZP had a small activation energy of 0.34 eV, indicating a fast Li-ion transfer in the NASICON framework.

**Figure 4:**
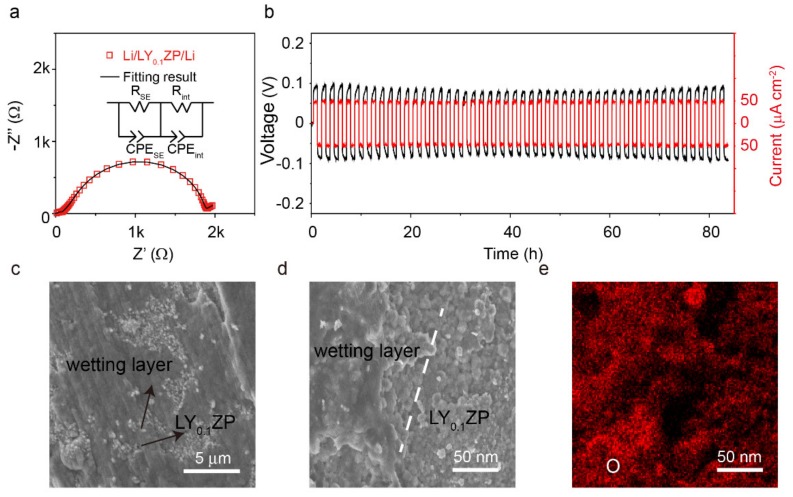
(**a**) Impedance plot of a symmetric Li/LY0.1ZP/Li cell tested at 60 °C; the applied frequency for the symmetric cell was 1 MHz–1 Hz. (**b**) The cycling stability of LY0.1ZP electrolytes in a symmetric Li/LY0.1ZP/Li cell at 0.05 mA cm^−2^ at 60 °C. (**c**) The top view and (**d**) cross-section images of LY0.1ZP after contacting with lithium metal. (**e**) The mapping of O element on cycled LY0.1ZP. Scale bar: 50 nm; the size of the Li foil was 0.5 cm^−2^.

The electrochemical stability of LY_0.1_ZP with lithium metal was studied by assembling in a symmetric cell. The LY_0.1_ZP electrolyte prepared by hot pressing shows an interfacial resistance with lithium metal of 392 Ω cm^2^ at 60 °C (Figure 4a), which is comparable with Ge-coated LAGP [51]. A fixed current density of 0.05 mA cm^−2^ was used to evaluate the reversibility of the Li plating and stripping process of LY_0.1_ZP in the symmetric cell. The resulting galvanostatic profile is shown in Figure 4b; a polarization voltage of 0.1 V with a negligible variation was observed within 80 h. The SEM images in Figure 4c,d exhibit a uniform wetting layer on LY_0.1_ZP after contact with lithium metal. The cross-section image and the EDS mapping of the LY_0.1_ZP in Figure 4e and Figure S6 show that the LY_0.1_ZP maintained good compactness, and the passivation product was a glassy phase that wet well on the LY_0.1_ZP surface. A glassy interphase layer leads to good contact between the electrode and electrolyte. The pathway of Li^+^ ions across the Li/NASICON interface is continuous, resulting in a small interfacial resistance. The mapping images confirmed that the interfacial area had a homogenous distribution of Zr, P, Li and O elements.

**Figure 5:**
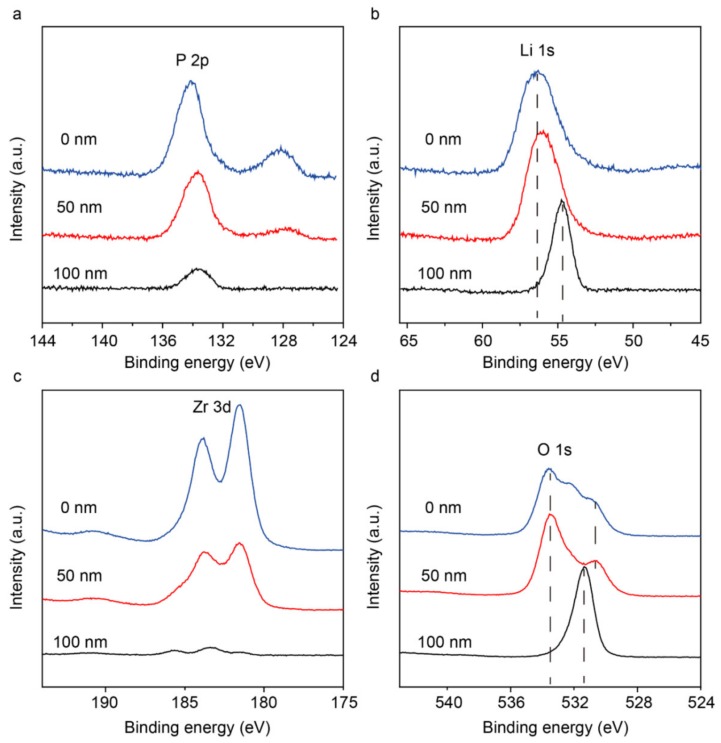
The XPS spectra of LY0.1ZP cycled in a Li/Li cell. The evolution profiles of relative (**a**) P, (**b**) Li, (**c**) Zr, (**d**) O elemental intensities along the depth direction. The separation distance was 50 nm, calculated from the Ar sputtering.

To further identify the composition distribution at the interface area, XPS depth profiles were collected. Figure S7 presents the atomic concentration distribution along with the depth from the top section. The content of O, Li, P, Zr elements in LY_0.1_ZP after cycling in the symmetric cell remained almost unchanged within the recorded depth, which agrees well with the EDS mapping results. Detailed analysis of the P 2p spectrum (Figure 5a) indicates that the top interface consists of PO_4_^3−^ (135 eV) and P^3−^ (129 eV) signals, which are contributed from PO_4_^3−^ tetrahedral and Li_3_P, respectively. The peak corresponding to Li_3_P disappeared after full removal of the top layer (–100 nm). LY_0.1_ZP reacts with lithium metal to form a Li_3_P layer with a thickness of 50 nm, and the reaction increases the contact between lithium metal and LY_0.1_ZP; the good ionic conductivity of the Li_3_P layer could help improve the wettability of the LY_0.1_ZP pellet by the metallic lithium anode. Figure 5b indicates the related shift of Li 1s peak. Peak splitting attributable to local an oxygen coordination change from bridging PO_4_^3−^ to nonbridging P-O-P was found in the O 1s spectra at 533 and 531 eV, indicating the P^4+^-O^2−^ bond breakage at the surface. Meanwhile, the peak at 531.6 eV is ascribed to the O^2–^ 2p binding energy in LY_0.1_ZP. The presence of P^3−^, the change of O 2s, and the Li 1s on the LY_0.1_ZP surface suggest the self-compatibility process of LY_0.1_ZP cycled in the Li/Li symmetric cell.

**Figure 6:**
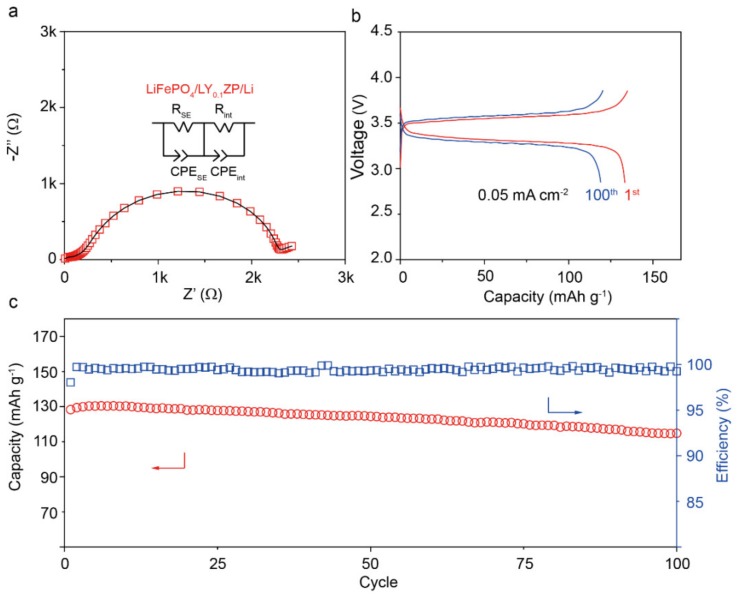
(**a**) Impedance profile of the all-solid-state LiFePO4/LY0.1ZP/Li cell at 60 °C. (**b**) Charge and discharge voltage profiles and (**c**) cycling performance of the all-solid-state LiFePO4/LY0.1ZP/Li cell at 60 °C. The size of the LiFePO4 was 0.5 cm^−2^.

The performance of LY_0.1_ZP in all-solid-state Li-metal batteries with a composite LiFePO_4_ cathode was studied. To further reduce the Li/LY_0.1_ZP interfacial resistance, and to improve the cycling performance of the cell, a Li-ion conducting polymer was put between the lithium metal anode and the LY_0.1_ZP solid electrolyte, which is similar to other reports [30]. The obtained Li/Polymer/LY_0.1_ZP/LFP solid-state cell had a total resistance of 2200 Ω at 60 °C (Figure 6a). Figure 6b is the charge/discharge voltage profile of the all-solid-state battery at current densities of 0.05 mA cm^−2^ (about 0.1C); the cell showed a flat voltage plateau during charge/discharge with a small overpotential of 0.15 V at 0.05 mA cm^−2^. The discharge capacity of the cell was 130 mAh g^−1^ and a 119 mAh g^−1^ capacity was retained after 100 cycles (Figure 6c). The all-solid-state cell had a high coulombic efficiency of 99.5% during cycling, indicating a stable LY_0.1_ZP/cathode interface.

## 4. Conclusions

Rhombohedral LiZr_2_(PO_4_)_3_ phase was stabilized by Y^3+^ doping, and the Li_1.1_Y_0.1_Zr_1.9_(PO_4_)_3_ showed a high density and a high Li-ion conductivity of 9 × 10^−5^ S cm^−1^ at room temperature, which is two orders magnitude higher than that of LiZr_2_(PO_4_)_3_. The absence of a phase transition of Li_1.1_Y_0.1_Zr_1.9_(PO_4_)_3_ from rhombohedral to triclinic at −30 °C showed the excellent structural stabilization by Y^3+^ doping. With the formation of a self-wetting layer at the LY_0.1_ZP/Li interface, highly reversible dendrite-free Li plating/stripping at a current density of 0.05 mA cm^−2^ was achieved. Moreover, the Li_1.1_Y_0.1_Zr_1.9_(PO_4_)_3_ pellet showed a relatively small interfacial resistance in symmetric Li/Li and all-solid-state Li-metal cells. The long-term cycling performance of the all-solid-state cell demonstrates that Li_1.1_Y_0.1_Zr_1.9_(PO_4_)_3_ prepared by hot-pressing is a promising candidate for all-solid-state Li-metal batteries.

## Supplementary Materials

The following are available online at https://www.mdpi.com/1996-1944/13/7/1719/s1, Figure S1: The lattice parameters of the sample with (**a**) different Y^3+^ concentration and (**b**) different temperatures, Figure S2: The refinement of the XRD data of NAISCON Li_1+x_Y_x_Zr_2-x_(PO_4_)_3_ (0.1 ≤ x ≤ 0.2). The green and yellow vertical lines are the Braga positions of the rhombohedral and triclinic phases, respectively. The red circle and the black line is the experimental data and the fitting result, respectively. The small chi2 value indicates a good fitting, Figure S3: Relative densities of Li_1+x_Y_x_Zr_2-x_(PO_4_)_3_ (0.1 ≤ x ≤ 0.2) pellets treated by regular sintering and hot-pressing from 1100 to 1250 °C, Figure S4: SEM images of LY_0.1_ZP pellets prepared by hot-pressing from 1100 to 1250 °C, Figure S5: Nyquist plots of Li_1+x_Y_x_Zr_2-x_(PO_4_)_3_ (0.1≤x≤0.2) pellets prepared by regular sintering and hot-pressing, Figure S6: EDS images of P, Zr, Y elements distribution on cycled LY_0.1_ZP in a Li/Li cell, Figure S7: Atomic percentages along with the depth of cycled LY_0.1_ZP in a Li/Li cell.
