# Supplementary material for: Rhombohedral Li1+xYxZr2-x(PO4)3 Solid Electrolyte Prepared by Hot-Pressing for All-Solid-State Li-Metal Batteries
Source: Materials (Basel). 2020 Apr 6;13(7):1719. doi: 10.3390/ma13071719 (PMC7178664; doi:10.3390/ma13071719)
Supplement: Supplementary file 1 [file materials-13-01719-s001.pdf]

materials

## Supplementary Materials

# Rhombohedral $Li_{1+x}Y_xZr_{2-x}(PO_4)_3$ Solid Electrolyte Prepared by Hot-Pressing for All-Solid-State Li-Metal Batteries

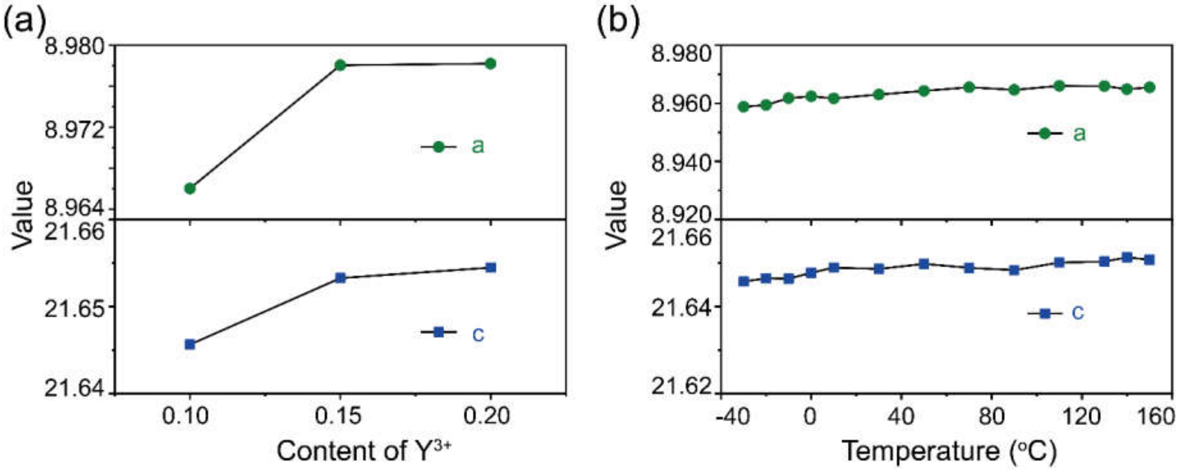


**Figure S1.** The lattice parameters of the sample with (**a**) different $Y^{3+}$ concentration and (**b**) different temperatures.

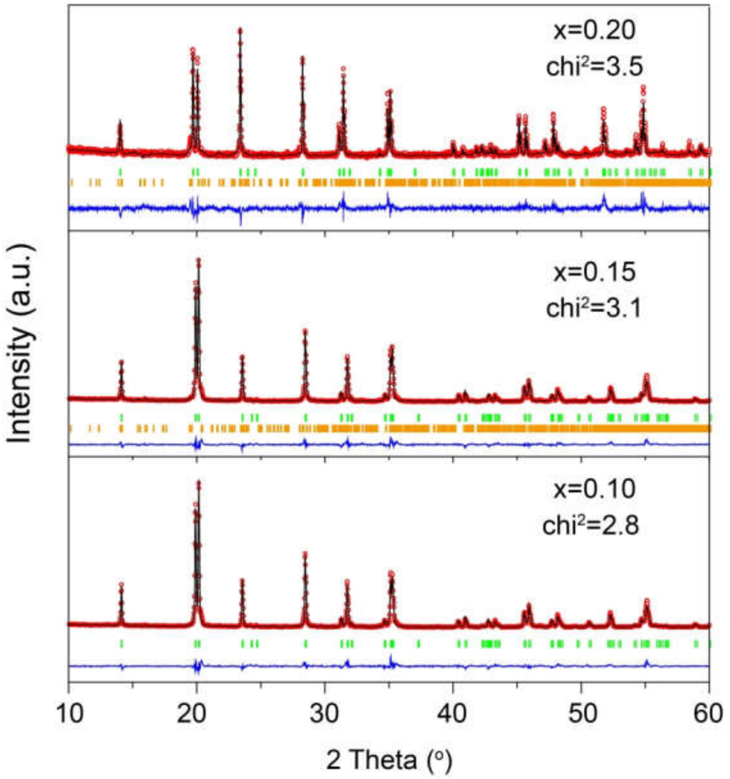


**Figure S2.** The refinement of the XRD data of NAISCON $Li_{1+x}Y_xZr_{2-x}(PO_4)_3$ ($0.1 \leq x \leq 0.2$). The green and yellow vertical lines are the Braga positions of the rhombohedral and triclinic phases,

respectively. The red circle and the black line is the experimental data and the fitting result, respectively. The small chi2 value indicates a good fitting.

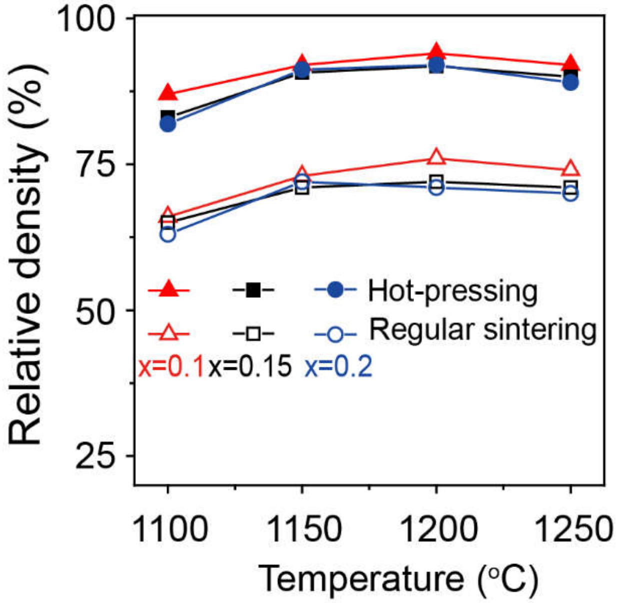


**Figure S3.** Relative densities of $Li_{1+x}Y_xZr_{2-x}(PO_4)_3$ (0.1 ≤ x ≤ 0.2) pellets treated by regular sintering and hot-pressing from 1100 to 1250 °C**.**

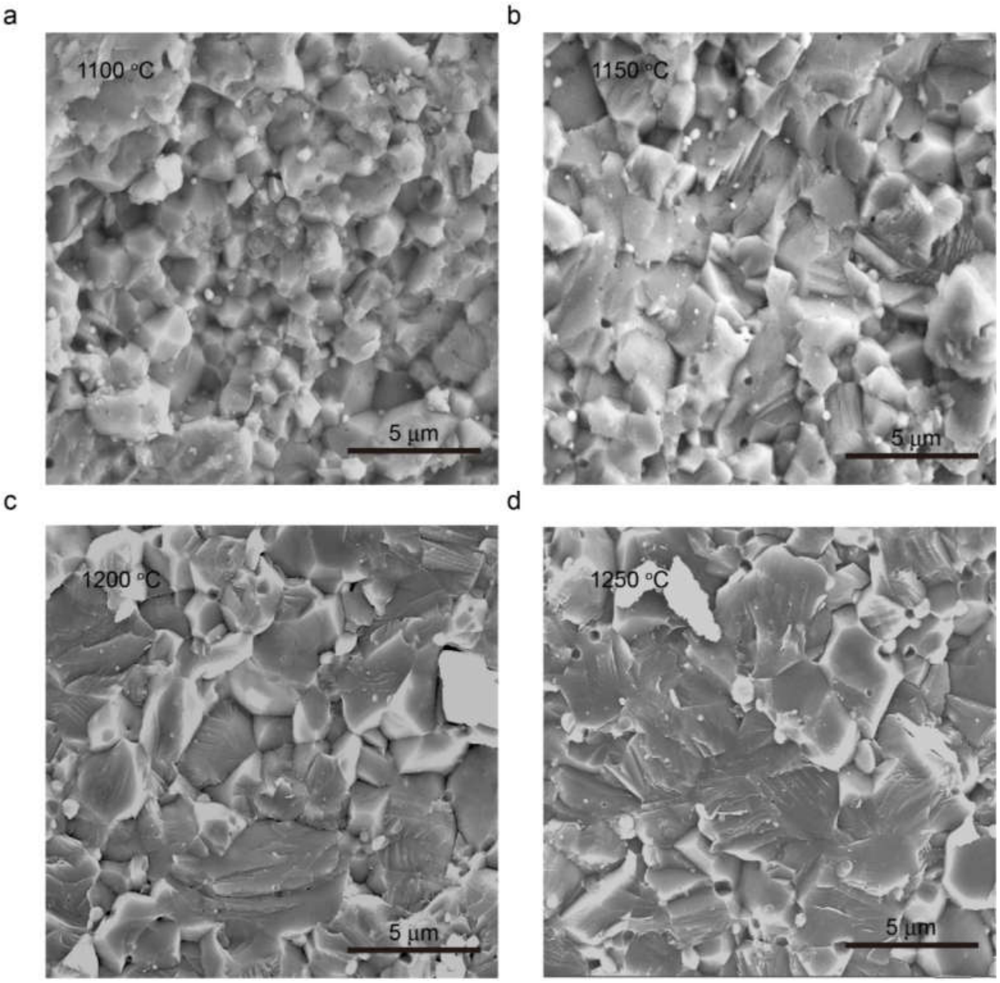


**Figure S4.** SEM images of LY$_{0.1}$ZP pellets prepared by hot-pressing from 1100 to 1250 °C.

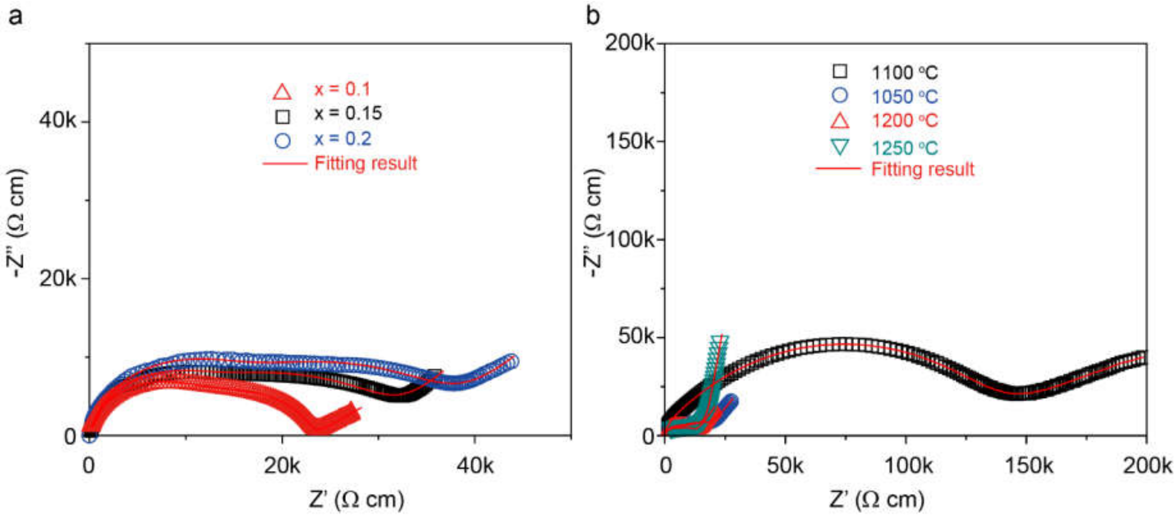


**Figure S5.** Nyquist plots of $Li_{1+x}Y_xZr_{2-x}(PO_4)_3$ (0.1≤x≤0.2) pellets prepared by regular sintering and hot-pressing.

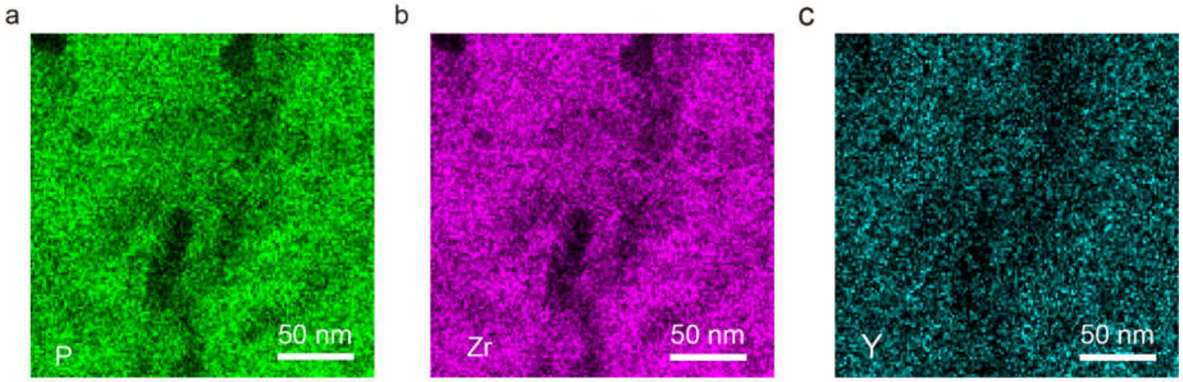


**Figure S6.** EDS images of P, Zr, Y elements distribution on cycled $LY_{0.1}ZP$ in a Li/Li cell.

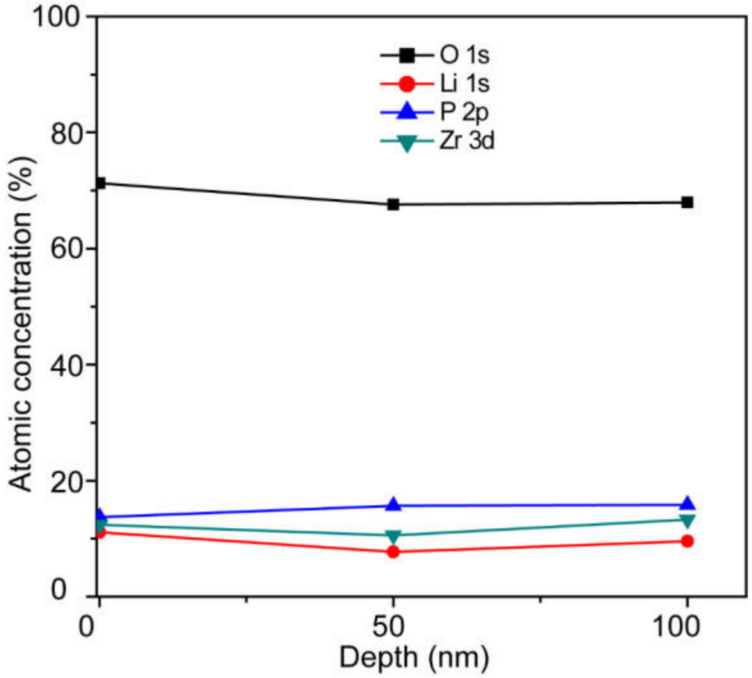


**Figure S7.** Atomic percentages along with the depth of cycled $LY_{0.1}ZP$ in a Li/Li cell.
